# Altitude and risk of pre-eclampsia: insights from a large-scale epidemiological study in Ecuador

**DOI:** 10.1098/rstb.2024.0169

**Published:** 2025-08-21

**Authors:** Emma Mitchell-Sparke, Bart Theeuwes, Imogen Grant, Dino A. Giussani, Catherine Aiken

**Affiliations:** ^1^ Department of Obstetrics and Gynaecology, University of Cambridge, Cambridge, UK; ^2^ Tufts University School of Medicine, Boston, MA, USA; ^3^ Cambridge Stem Cell Institute, University of Cambridge, Cambridge, UK; ^4^ Department of Physiology, Development, and Neuroscience, University of Cambridge, Cambridge, UK; ^5^ Institute of Metabolic Science, University of Cambridge, Cambridge, UK; ^6^ Loke Centre for Trophoblast Research, University of Cambridge, Cambridge, UK; ^7^ Cambridge Reproduction Strategic Research Initiative, University of Cambridge, Cambridge, UK

**Keywords:** pre-eclampsia, altitude, Ecuador, maternal health, socioeconomic status, generalized additive models

## Abstract

Exposure to hypobaric hypoxia at high altitude is associated with adverse pregnancy outcomes, including low birth weight and stillbirth. However, the relationship between high altitude and pre-eclampsia remains less well defined, with conflicting findings in previous studies. Improved understanding of pre-eclampsia risk at altitude could help in targeting resources, particularly in low-resourced settings. This study aimed to evaluate the impact of altitude, analysed as a continuous nonlinear variable, on the risk of pre-eclampsia in Ecuador. Publicly available maternal health records from Ecuador (2021–23) were obtained from the National Institute of Statistics and Census. Geospatial techniques were used to assign the altitude of maternal residence, and generalized additive models determined the relationship between altitude and pre-eclampsia incidence, adjusting for maternal age, ethnicity, healthcare access, and residence. Models were fitted using restricted maximum likelihood. After adjusting for confounders, no significant increase in pre-eclampsia risk was observed with higher altitude. However, as expected, increased risk was noted at the extremes of maternal age (*p *<0.001) and among women with publicly funded care (*p *<0.001). Ethnicity was also a risk determinant, but no interaction with altitude was found. Comparison of our findings with population-wide studies elsewhere is a key future goal.

This article is part of the discussion meeting issue ‘Pregnancy at high altitude: the challenge of hypoxia’.

## 1. Introduction

Pre-eclampsia impacts 2–8% of pregnancies globally. It remains a major cause of maternal and fetal mortality worldwide [1], although the majority of pre-eclampsia-related deaths occur in low- and middle-income countries [2]. Pre-eclampsia is diagnosed by new-onset hypertension (defined as systolic blood pressure ≥140 mmHg and/or a diastolic blood pressure ≥90 mmHg) accompanied by signs of maternal end-organ damage (e.g. proteinuria) [3]. This condition occurs most often after 20 weeks of gestation and frequently near term. Understanding how pre-eclampsia risk varies across global populations enables new insights into aetiology and appropriate targeting of clinical resources. This is particularly important in settings where pre-eclampsia risk is high and maternity care may be under-resourced.

Pre-eclampsia originates from defective invasion of extravillous trophoblast into the maternal spiral arteries, leading to incomplete spiral artery remodelling and thus altered placental perfusion [1,4]. Altered perfusion leads to dysfunctional placental energy metabolism, with consequent redox imbalance, oxidative stress, and altered gene expression [5]. The resultant widespread endothelial activation within the maternal circulation contributes to the diverse range of clinical manifestations of pre-eclampsia, including acute renal injury, strokes, seizures, pulmonary oedema, and death [4,6].

There are well-described risk factors associated with the development of pre-eclampsia, e.g. nulliparity, chronic hypertension, extremes of maternal age, and low socio-economic status [7,8]. By contrast, a relatively understudied potential risk factor is the impact of high altitude on pre-eclampsia [9–12]. It is hypothesized that exposure to hypobaric hypoxia at high altitude may be synergistic with the physiological challenges of pregnancy to the maternal cardiovascular system [10–12]. Structural and functional changes in placentas exposed to high-altitude hypoxia have been observed, including vascular alterations and increased nutrient transport capacity [13,14], leading to the hypothesis that hypobaric hypoxia may impair spiral artery remodelling, resulting in a higher risk of pre-eclampsia [14].

However, previous studies of the relationship between altitude and pre-eclampsia risk show mixed results [9]. Recently, a large state-wide study from Colorado has shown that exposure to high altitude, defined as higher than 2500 m, increases the risk of pre-eclampsia by around 33% compared to the population residing at altitudes of less than 2500 m [15]. However, a study of births across Ecuador between 2015 and 2017 showed a variable effect of altitude on pre-eclampsia risk, with decreased risk observed at moderately high altitudes (1500−3500 m) and increased risk noted at extremely high altitudes (>3500 m) [11]. These mixed findings suggest that the effect of altitude on pre-eclampsia risk may (i) not be linear and/or (ii) depend on context-specific factors.

In this study, we investigate the relationship between altitude and pre-eclampsia risk across Ecuador using the most recent data available. We take a non-parametric approach to altitude (treated as a continuous variable), correcting for available confounding variables. Such nonlinear modelling avoids prior assumptions about the relationship between altitude and pre-eclampsia risk, as well as arbitrary binning of altitude levels. Together, this approach allows for a more nuanced understanding of how incremental changes in altitude influence pre-eclampsia risk, providing critical insights relevant to both health policy and clinical care.

## 2. Methods

### (a) Dataset

Data regarding all hospital discharges across Ecuador during 2021−2023 (inclusive) was filtered to include only obstetric admissions. We then selected all women who had given birth and divided them by three-letter International Classification of Diseases-10 (ICD-10) codes into those who had experienced pre-eclampsia, defined as any of O11 (superimposed pre-eclampsia), O14 (pre-eclampsia) or O15 (eclampsia), versus those who had other obstetric coding but no evidence of pre-eclampsia. The dataset includes women who gave birth in settings that report data to the National Institute of Statistics and Census of Ecuador; thus, women who gave birth elsewhere (e.g. at home) are not represented.

### (b) Geocoding and altitude coding

Altitude (metres) of normal residence of each pregnant woman was the exposure variable in the analysis. In our study, the altitude range of residence for participants spanned from approximately sea level (−21 m) to 4436 metres above sea level (highest recorded parish altitude in dataset). The middle 50% of patients lived at altitudes between 10 and 2129 m. A small minority, approximately 3.87% of participants (*n* = 19 190), resided at altitudes above 3000 m. For a clearer understanding of the geographical diversity and distribution of the study population, see the electronic supplementary material, figure S1, which displays the percentage of women across altitude levels in this Ecuador dataset.

**Figure 1.**
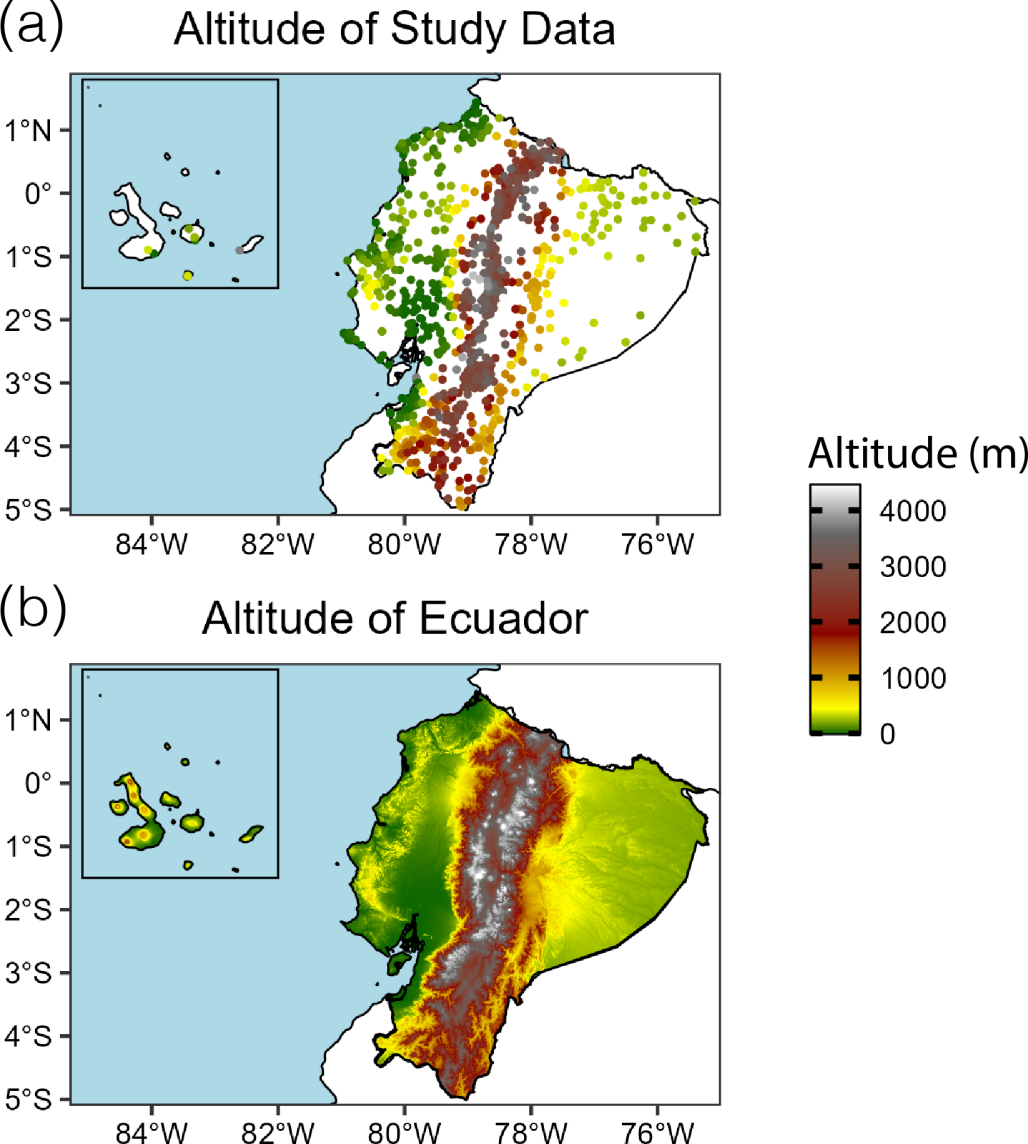
(a) Ecuador study data points plotted by calculated altitude and (b) Ecuador terrain map (data from AWS via ‘elevatr’ package).

Residence was coded at the level of ‘parish’, the smallest geographical administrative unit in Ecuador. There is significant variation in the sizes of parishes in Ecuador, some of which cover very large areas, especially in rural regions. An example of a small size of parish is around 16 km^2^. To determine altitude, each parish was geocoded to acquire latitude and longitude coordinates using the Google Maps API service. These coordinates were then used to retrieve altitude using the ‘elevatr’ package, sourcing the data from Amazon Web Services Terrain Tiles. To verify the accuracy of our geocoding, we created a scatterplot of the parish coordinates, colour-coded by altitude. This map was then visually compared to an Ecuador terrain map, also generated in R using the ‘elevatr’ package. The computed altitude data aligned with known geographical patterns, confirming that our data accurately represents the topographical variations across Ecuador (figure 1a,b).

### (c) Variables and analysis

Pre-eclampsia was coded by clinical providers according to local definitions and labelled as ‘pet’ in the dataset. We categorized the funding model into private, non-profit, and public care sectors. Healthcare facility type was recoded into four categories: clinics, basic hospitals, general hospitals, and specialist care. Urban versus rural residence was coded as a binary variable. Maternal age was treated as a continuous numeric variable (refer to the electronic supplementary material, figure S2, for a visual representation of maternal age distribution across this dataset).

Self-reported ethnicity was recoded into broader categories: individuals who identified as ‘Afroecuatoriano/a Afrodescendiente’, ‘Mulato/a’ or ‘Negro/a’ were classified as ‘Afro-Ecuadorian’; ‘Mestizo/a’ was recoded to ‘Mestiza’; ‘Blanco/a’ was simplified to ‘White’; ‘Indígena’ was categorized as ‘Andean’; and ‘Montubio/a’ was recoded to ‘Montubian’, referring to those members of the population who originally dwelt in coastal regions and later admixed with Spanish settlers and Africans [16]. Those without clear ethnicity information (‘Ignorado/a’, ‘Sin información’ or ‘Otro/a’) were grouped as ‘other’. These groupings are recognized by the Ecuadorian census as the five major ethnic groups in Ecuador [16]. We further classified women according to whether they were of Ecuadorian or other nationality as a binary variable; of 495 797 women, greater than 99% were Ecuadorian or from a neighbouring state (Venezuela, Colombia, Peru); therefore, no exclusions were made on the basis of nationality. Lastly, we examined the impact of birth month on risk of pre-eclampsia, and, having found no significant effect in our models’ predictions, we dropped this variable from model analyses.

We explored the association between variables of interest and pre-eclampsia risk using chi-squared or Student’s *t*‐test (two-sided). We used unadjusted and adjusted linear, generalized linear and additive dynamic models as appropriate to summarize the relationships between key variables of interest. Where parametric models were specified, we estimated the effect of 500 m increases in altitude to improve interpretability. The adjusted models included maternal age, ethnicity, funding model, medical type of care, and urban versus rural residence as covariates. The model’s goodness-of-fit was evaluated using the Akaike information criterion.

For the primary analysis, pre-eclampsia risk was modelled as a binary outcome with altitude as a smoothed independent variable using an adjusted binomial model fitted via restricted maximum likelihood. Generalized additive modelling (GAM) was used to allow non-parametric model fitting with relaxed assumptions about the functional form of the relationship between pre-eclampsia risk and altitude. Models were constructed by iteratively fitting weighted additive models through backfitting. Our models specified a nonlinear term for the effect of altitude, estimated using cubic splines. The backfitting algorithm was a Gauss-Seidel method for fitting additive models by iteratively smoothing partial residuals. Confidence intervals (CIs) were included in plots to convey uncertainty in the model estimates. GAM modelling was essential owing to the potential nonlinear or absent relationship between altitude and pre-eclampsia, given that biological variables often do not exhibit linear relationships.

All data processing and statistical analyses were conducted in R v. 4.4.1 (R Core Team, 2024), and results were reported as odds ratios with 95% CIs with two-sided *p*-values. The study adhered to the Strengthening the Reporting of Observational Studies in Epidemiology guidelines for reporting.

### (d) Ethics statement

The dataset used in this analysis was obtained from the publicly available source provided by the National Institute of Statistics and Census of Ecuador (https://www.ecuadorencifras.gob.ec/camas-y-egresos-hospitalarios/). Since all data used was freely and publicly available and fully anonymized, the study did not require Institutional Review Board or ethical approval. The data and accompanying data dictionary, originally in Spanish, were translated into English during analysis.

## 3. Results

During the study period (2021−2023), 495 797 women who gave birth in Ecuador were included in the analytic sample. Pre-eclampsia was diagnosed in 4.75% of cases (95% CI 4.69−4.81; *n* = 23 537 out of 495 797). Average maternal age was 26.41 ± 6.66 in patients without pre-eclampsia and 27.02 ± 7.25 in patients with pre-eclampsia (table 1).

**Table 1.** Study population characteristics, including ethnicity, type of care, residence, funding model, and maternal age, with chi-squared values and corresponding *p*-values reported. (Asterisk shows association with *p*-value <0.05, denoting statistical significance.)

	patients without pre-eclampsia % (*n*)	patients with pre-eclampsia % (*n*)	significance testing chi-squared (*p*‐value)
**total**	95.3 (472 260)	4.8 (23 537)	N/A
**ethnicity**			
Afro-Ecuadorian	1.6 (7429)	2.8 (666)	219.6 (0.00*)
Mestiza	87.5 (413 272)	86.8 (20 434)	9.8 (0.00*)
Montubia	0.3 (1443)	0.3 (76)	0.2 (0.68)
Andean	5.5 (25 880)	4.6 (1080)	34.5 (0.00*)
White	0.4 (1722)	0.3 (65)	4.6 (0.03*)
other	4.8 (22 514)	5.2 (1216)	7.8 (0.01*)
**type of care**			
clinic	10.3 (48 501)	1.4 (336)	1973.1 (0.00*)
basic hospital	39.2 (185 248)	16.5 (3893)	4889.1 (0.00*)
general hospital	41.6 (196 591)	69.5 (16 359)	7108.7 (0.00*)
specialist care	8.9 (41 920)	12.5 (2949)	363.0 (0.00*)
**residency**			
urban	79.9 (377 168)	79.3 (18 653)	5.2 (0.02*)
rural	20.1 (95 092)	20.8 (4884)	5.2 (0.02*)
**funding model**			
private	26.5 (125 004)	6.0 (1422)	4923.6 (0.00*)
not-for-profit (nfp)	2.0 (9349)	0.3 (79)	323.9 (0.00*)
public	71.6 (337 907)	93.6 (22 036)	5489.2 (0.00*)
**maternal age** mean (s.d.)	26.4 (6.66)	27.0 (7.25)	0.00*

**Figure 2.**
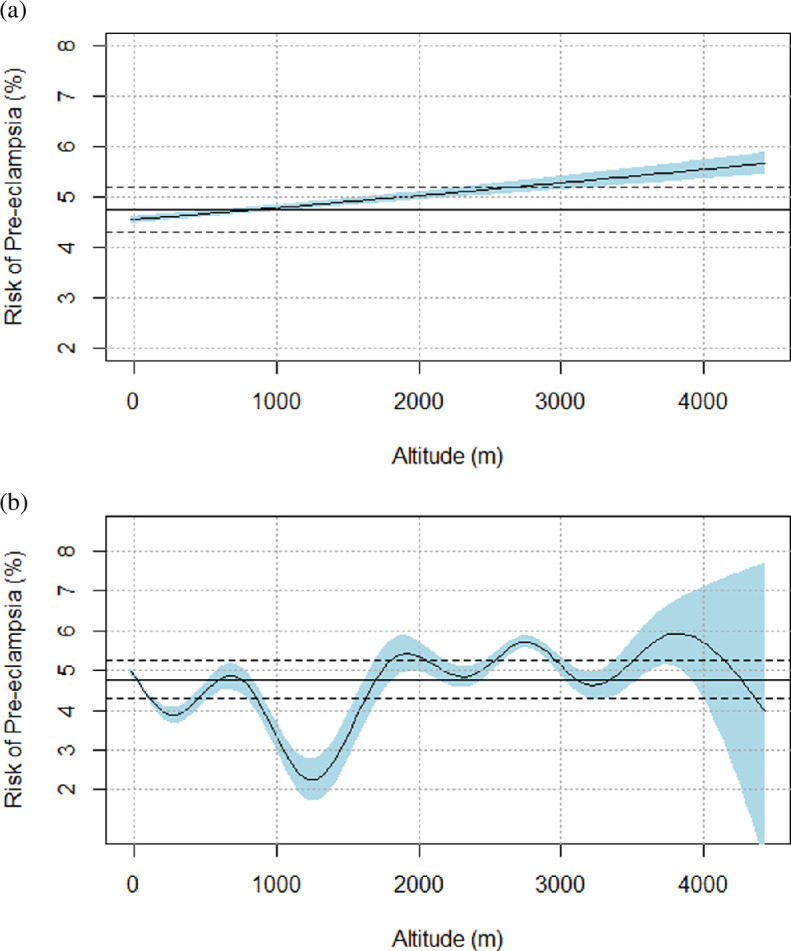
(a) Risk of pre-eclampsia versus altitude: modelled as an unadjusted continuous linear variable. (b) Risk of pre-eclampsia versus altitude: modelled as an unadjusted continuous non-parametric variable.

When altitude was modelled as an unadjusted linear variable, the odds of developing pre-eclampsia increased by 2.6% above baseline risk (<0.1% absolute risk increase) per 500 m (figure 2a). In unadjusted non-parametric analysis, the relationship between altitude and pre-eclampsia was nonlinear and remained relatively constant above 1800 m (figure 2b).

We proceeded to identify demographic characteristics that were associated with pre-eclampsia risk. Calculations of the chi-squared comparisons across these characteristics between the pre-eclampsia and non-pre-eclampsia groups, with corresponding statistical significance denoted in *p*-values, are reported in table 1. Of the study population, 72.6% delivered in public hospitals versus 27.4% with private or non-profit care (table 1). Publicly funded women experienced more than five-fold higher pre-eclampsia risk compared to those delivering in private care (table 1). Approximately 20.2% of women were residing in rural areas (table 1). The risk of pre-eclampsia was relatively similar between women living in rural versus urban areas, found to be 4.89 and 4.71, respectively (table 1). Referral to specialist obstetric care and care in general hospitals were associated with a higher increase in pre-eclampsia risk compared to delivery in clinic or basic hospital settings (table 1). When calculated by ethnic groupings, we found that compared to Mestizan women, Afro-Ecuadorian women experienced a 1.74-fold increase in risk, while Andean women had decreased risk (table 1). For visual representations of these characteristics impact on pre-eclampsia (reported as a percentage of the population), refer to the electronic supplementary material, figures S6–S8.

**Figure 3.**
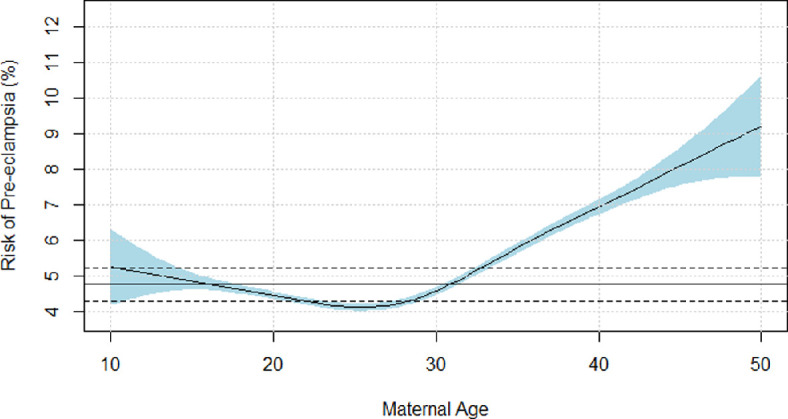
Risk of pre-eclampsia versus maternal age: modelled as a continuous non-parametric variable, adjusted for maternal age, type of care, funding model, urban versus rural residence, and ethnicity.

**Figure 4.**
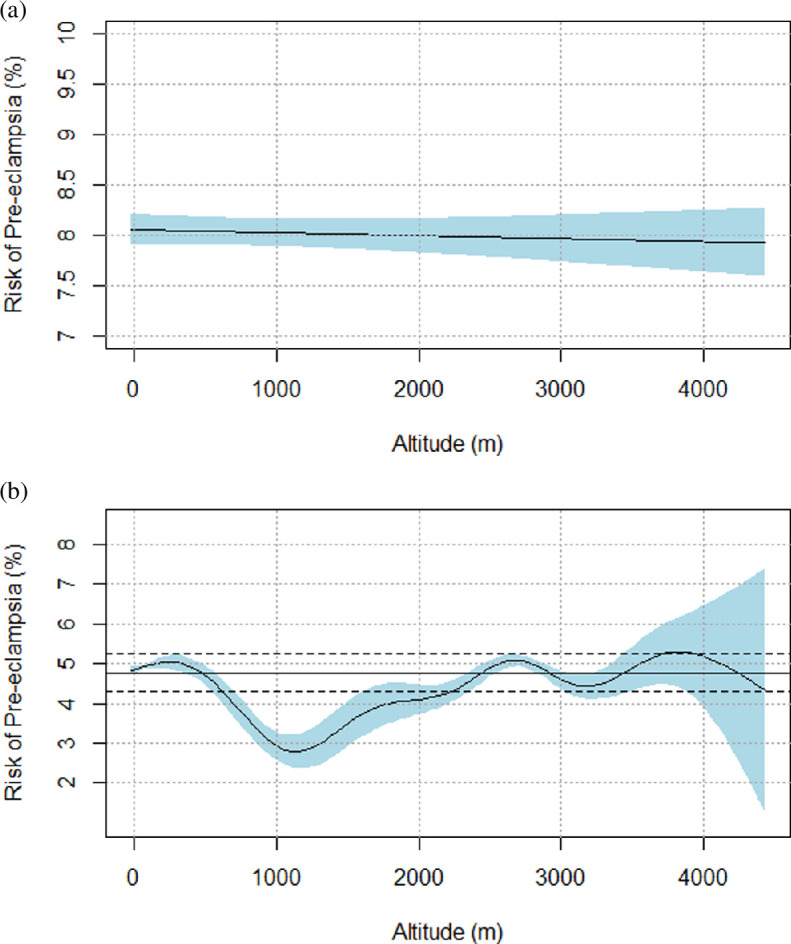
(a) Relative risk of pre-eclampsia versus altitude: modelled as a continuous linear variable, adjusted for maternal age, type of care, funding model, urban versus rural residence, and ethnicity. (b) Risk of pre-eclampsia versus altitude: modelled as a continuous non-parametric variable, adjusted for maternal age, type of care, funding model, urban versus rural residence, and ethnicity.

**Figure 5.**
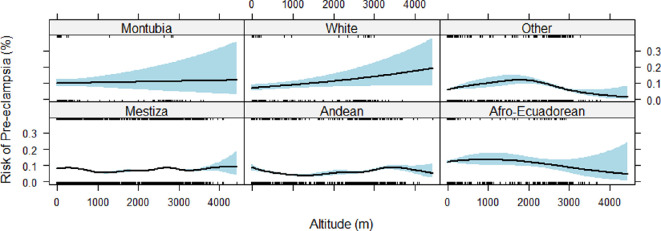
Risk of pre-eclampsia versus altitude by ethnicity: modelled as a continuous non-parametric variable, adjusted for maternal age, type of care, funding model, urban versus rural residence, and ethnicity.

After adjustment for relevant covariates, factors that influenced pre-eclampsia risk were maternal age, type of care, ethnicity, and funding model (table 2). Specifically, the incidence of pre-eclampsia was more common at the extremes of maternal age, either less than 17 years old or more than 32 years old (figure 3), compared to average incidence. After adjustment, we found no evidence of altitude affecting the risk of pre-eclampsia in either linear (figure 4a) or non-parametric (figure 4b) analysis. While ethnicity-related differences in pre-eclampsia risk persisted in adjusted modelling, we found no evidence of an interactive effect between ethnicity and altitude on pre-eclampsia risk (figure 5).

**Table 2.** Risk of developing pre-eclampsia odds ratios and 95% confidence intervals derived from unadjusted and adjusted generalized linear models. (There was no substantive difference between these values and those derived from models where altitude was modelled as a non-parametric function. Adjusted models include maternal age, funding model, type of care, ethnicity, and residence. Asterisk shows association with *p*-value <0.05, denoting statistical significance.)

	unadjusted pre-eclampsia or [lower CI, upper CI] *p*‐value	adjusted pre-eclampsia or [lower CI, upper CI] *p*‐value
**ethnicity**		
Mestiza	ref	ref
Afro-Ecuadorian	1.8 [1.7, 2.0] 0.00*	1.6 [1.5, 1.7] 0.00*
Montubia	1.1 [0.8, 1.3] 0.59	1.3 [1.0, 1.6] 0.05
Andean	0.8 [0.8, 0.9] 0.00*	0.7 [0.7, 0.8] 0.00*
White	0.8 [0.6, 1.0] 0.03*	1.0 [0.8, 1.3] 0.91
other	1.1 [1.0, 1.2] 0.00*	0.8 [0.8, 0.9] 0.00*
**type of care**		
clinic	ref	ref
basic hospital	3.0 [2.7, 3.4] 0.00*	1.3 [1.1, 1.4] 0.00*
general hospital	12.0 [10.8, 13.4] 0.00*	4.0 [3.5, 4.5] 0.00*
specialist care	10.2 [9.1, 11.4] 0.00*	4.1 [3.6, 4.6] 0.00*
**residency**		
urban	ref	ref
rural	1.0 [1.0, 1.1] 0.02*	1.0 [1.0, 1.1] 0.06
**funding model**		
private	ref	ref
not-for-profit (nfp)	0.7 [0.6, 0.9] 0.01*	0.5 [0.4, 0.6] 0.00*
public	5.7 [5.4, 6.1] 0.00*	3.3 [3.1, 3.5] 0.00*
**maternal age**	1.0 [1.0, 1.0] 0.00*	1.0 [1.0, 1.0] 0.00*
**altitude (m)**		
<1000	ref	ref
1000−2000	0.81 [0.74, 0.87] 0.00*	0.72 [0.67, 0.79] 0.00*
2000−3000	1.18 [1.15, 1.22] 0.00*	1.00 [0.97, 1.03] 0.87
>3000	1.11 [1.04, 1.18] 0.00*	0.97 [0.90, 1.04] 0.37

## 4. Discussion

Our findings suggest that, in the contemporary Ecuadorian context, the risk of pre-eclampsia does not increase at higher altitudes. Instead, the strongest predictors of developing pre-eclampsia are publicly funded healthcare status and extremes of maternal age. Given that publicly funded compared with privately funded healthcare status could be considered a proxy for socio-economic status, our results are in keeping with previous work [3,8,11], suggesting that pre-eclampsia is more common among lower-resourced women.

Other adverse pregnancy outcomes, such as stillbirth and reduced birth weight, appear to be consistently increased at higher altitudes [9,17–19]. There is also evidence that blood pressure is elevated in high-altitude pregnancies (>2500 m) compared to those at lower altitudes (<1500 m) across global settings [9]. However, the magnitude of this increase is minimal, and there is less clarity about how this translates to hypertensive disorders of pregnancy [9]. For the specific outcome of pre-eclampsia, in keeping with previous studies [11,12,20], we did detect a marginal increase in risk at higher altitudes in unadjusted models. However, this relationship was not robust after adjustment for other relevant factors, suggesting that it was explained more by the variation in other parameters (e.g. socio-economic status) in the higher altitude population, rather than by the physiological impacts of high altitude on pregnancy. For visual representations of the interactions between altitude and pre-eclampsia across other background factors (e.g. funding model, type of care, ethnicity), refer to the electronic supplementary material, figures S3–S5. These figures demonstrate the evident impact of socio-demographic factors on pre-eclampsia risk with increasing altitude, indicating that what prior studies may have attributed to rising altitude levels may have more to do with socio-economic status.

Our results do not exclude the possibility of an underlying relationship between altitude and pre-eclampsia in other contexts or populations [15]. It has previously been suggested that Andean ethnicity may confer a reduced risk of low birth weight and pre-eclampsia at high altitude when compared with women of European ancestry, potentially via protective genetic polymorphisms [18,19,21–23]. However, while Andean women had lower rates of pre-eclampsia than women of other ethnicities in our dataset, there was no statistically significant interaction between ethnicity and altitude in determining pre-eclampsia risk.

Our finding that lower socio-economic status correlates with increased pre-eclampsia risk has also been observed in other global settings [8,24,25]; however, this relationship is poorly understood. It is uncertain whether the increased pre-eclampsia risk in women with socio-economic challenges may be owing to overall poorer health status, and hence an increased risk of disease states more generally, or to any related feature of disadvantage (e.g. poor diet [26]) that increases the likelihood of developing pre-eclampsia specifically. The increased risk of pre-eclampsia in lower-resourced settings could also be attributed to less access to appropriate medical care. Future research should focus on carefully disentangling multiple facets of socio-economic status that may be correlated with pre-eclampsia risk, particularly in low- and middle-income settings where resources are constrained and disadvantages magnified.

Our large-scale epidemiological study in Ecuador has significant advantages inherent in the study design. Our considerable sample size of nearly half a million women enhances the robustness of our findings and allows sufficient power to interrogate interactive relationships and fit powerful and flexible statistical models. The wide variation in altitude of Ecuador, ranging from sea level to over 4000 m, further allows for a comprehensive analysis of altitude-related effects on pre-eclampsia and other factors. Lastly, it is important to note that the recent nature of the data—collected from 2021 to 2023—enhances the relevance of our findings to current clinical contexts.

There are several key considerations in interpreting these findings. One important factor is the lack of precise address information for participants, which required the use of parish of residence and corresponding elevation data to approximate patients’ residential areas. As discussed in §2, parish sizes in Ecuador can vary significantly, with some rural parishes spanning large areas and encompassing a wide range of altitudes, potentially introducing variability in altitude measurements. Additionally, because the publicly available data included only women who gave birth in healthcare settings that report to the government, the dataset does not capture those who delivered elsewhere (e.g. at home); however, this group represents a minority at around 20% of the total population giving birth [27]. Given findings from prior Ecuadorian studies and census data, this group may differ in meaningful ways from the study population, as home births in Ecuador are disproportionately more common among Indigenous communities and lower-resourced populations [27,28]. As a result, our findings may be most applicable to women delivering in formal healthcare settings, and further research is needed to explore broader population trends.

Another aspect to consider is that only a small proportion of women in the dataset (3.87%) resided at particularly high altitudes (>3000 m). Consequently, findings at these extreme elevation levels should be interpreted with some caution, as the smaller sample size may affect the reliability of conclusions drawn for this subgroup. A further limitation of the study is that the dataset lacked specific measurements of socio-economic status (e.g. household income), which would be valuable for assessing the complex interplay between socio-economic factors and pre-eclampsia risk. Similarly, key covariates such as parity and body mass index [29] were not available, and these should be examined in future studies to provide a more comprehensive understanding of how altitude interacts with various maternal health factors.

It is important to acknowledge that different studies may have classified pre-eclampsia using varying criteria, particularly over time, which could affect direct comparisons with past research. Lastly, the retrospective nature of the study design carries inherent methodological challenges. Prospective or longitudinal studies incorporating genetic markers and detailed socio-economic data would thus provide important insight into the ways that genetic, social, and environmental factors shape pre-eclampsia risk at varying altitudes.

## 5. Conclusions

Pre-eclampsia risk in Ecuador is driven primarily by extremes of maternal age, specialist rather than general maternity care, and healthcare funding model. Close investigation of these relationships is likely to produce valuable further insights into the population-wide risks of pre-eclampsia. Importantly, we did not observe an association between increasing altitude and higher rates of pre-eclampsia in the Ecuadorian population. These findings must be carefully contextualized, not only to account for Ecuador’s unique ethnic diversity and topographic variation, but also to serve as a critical foundation for cross-country comparisons. Further research is essential to understand how pre-eclampsia risk manifests in other national contexts with varying socio-economic strata and geographic terrain in order to deepen our global understanding and guide more targeted interventions. 

## Data Availability

All supplementary data and figures presented in this manuscript were generated using R. The initial datasets used were obtained from the National Institute of Statistics and Census (INEC) of Ecuador, is publicly available at https://www.ecuadorencifras.gob.ec/camas-y-egresos-hospitalarios-informacion-historica/ and https://www.ecuadorencifras.gob.ec/camas-y-egresos-hospitalarios/. The R code used for the analysis and figure generation is available in the electronic supplementary material. The two input files for final data analysis are also included in the electronic supplementary material [30].
